# The nursing work environment and quality of care: Content analysis of comments made by registered nurses responding to the Essentials of Magnetism II scale

**DOI:** 10.1002/nop2.268

**Published:** 2019-04-09

**Authors:** Titilayo O. Oshodi, Benjamin Bruneau, Rachel Crockett, Francia Kinchington, Shoba Nayar, Elizabeth West

**Affiliations:** ^1^ Faculty of Health, Education, Medicine, and Social Care Anglia Ruskin University Chelmsford UK; ^2^ Faculty of Education and Health University of Greenwich London UK; ^3^ Division of Psychology, Faculty of Natural Sciences University of Stirling Stirling UK

**Keywords:** content analysis, Essentials of Magnetism II scale, nurses, nursing work, nursing work environment, quality of care, teamwork, ward manager support

## Abstract

**Aim:**

To report a qualitative study of themes Registered Nurses raised spontaneously about their work environment, in a cross‐sectional survey study when responding to the Essentials of Magnetism II (EOMII) scale.

**Design:**

Qualitative descriptive survey.

**Methods:**

At the end of the EOMII scale, a free form text section was included asking nurses to add comments about their ward/work environment*. *Of the 247 nurses who completed the EOMII scale, 30% (*N* = 75) provided comments. Inductive content analysis was used to analyse the textual information generated.

**Results:**

Three key themes emerged: “nurses need nurses to nurse”; working as a team and workplace environment. Participants described issues they were facing which comprised high turnover rates, inadequate staffing levels, increasing workload and high stress levels. Particular attention was drawn to the role of the ward manager in promoting a positive work environment, good teamwork and quality patient care.

**Impact Statement**

- Findings from this study have provided better understanding of the challenges experienced by nurses in their work environment, particularly in terms of constraints on their ability to provide high quality patient care.
- This study highlights the ward manager as a key determinant in nurses’ decisions to leave or to remain in the job, and makes evident the impact of the ward manager’s behaviour on staff morale.
- Findings from this paper should be an impetus for nurse leaders and policy makers to involve ward nurses in decision‐making and policies for practice.

## 1 INTRODUCTION

The Essentials of Magnetism II (EOMII) scale was developed in the United States by Schmalenberg and Kramer (2008), based on the characteristics of Magnets hospitals and was designed to measure healthy, attractive and productive, clinical work environments. Magnet hospitals define hospitals that attract and retain highly skilled professional nurses by providing positive working environment which promotes high job satisfaction for nurses and excellent patient care (Schmalenberg & Kramer, 2008). Magnet accreditation (American Nurses Credentialing Center [ANCC] (2017)) currently provides the only system for benchmarking nursing internationally without an equivalent alternative, although Health Education England (2016) is currently working with the Florence Nightingale Foundation to explore how the nursing excellence standards developed by the ANCC can be applied in England to promote learning and excellence in health and care practice.

**Figure 1:**
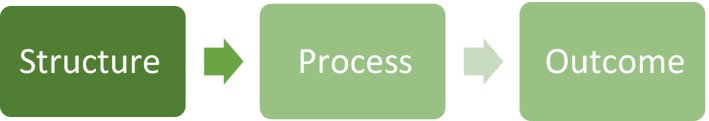
Structure–process–outcome model (adapted from Donabedian, 1980: 83)

The EOMII is a 58‐item four‐point, rating scale based on the process element of the Donabedian's (1992) Structure–Process–Outcome model. This model is linear and presumes that structure affects process and process, in turn, affects outcome (Donabedian, 1980), as shown schematically in Figure 1. The EOMII scale measures the following eight attributes: (a) nurse–physician relationships; (b) clinical autonomy; (c) patient‐centred culture; (d) working with clinically competent co‐workers; (e) control of nursing practice; (f) perceived adequacy of staffing; (g) support for education and (h) nurse manager support. The EOMII scale can facilitate investigation of the extent to which the work environment supports or hinders nurses in providing high quality patient care. It has been widely used in the United States and countries outside the United States, such as Turkey (Yildirim, Kisa, & Hisar, 2012), The Netherlands (De Brouwer, Kaljouw, Kramer, Schmalenberg, & Achterberg, 2014) and China (Bai et al., 2015). For the first time, in our earlier study (Oshodi, Crockett, Bruneau, & West, 2017), the EOMII was used in England.

## 2 BACKGROUND

The EOMII scale was distributed to 438 eligible Registered Nurses providing direct adult patient care in 29 wards in two NHS hospitals between May–October 2012. The EOMII scale was used in this study to address questions about the factor structure of the scale and to examine the associations, if any, between the factors and nurse‐assessed care quality. In our earlier study (Oshodi, Crockett, Bruneau, & West, 2017), the findings from this analysis were reported. On the survey questionnaire used in the above study, Registered Nurses were invited to write comments about their ward/work environments. In this paper, the qualitative analysis performed on this spontaneously generated free text data is reported.

## 3 THE STUDY

### 3.1 Aim

The purpose of this study was to identify Registered Nurses’ perceptions of and the quality of care in their working environment, by analysing the qualitative free text data provided by the nurses whilst responding to the EOMII scale.

### 3.2 Design

Qualitative description was the methodology of choice for this study. The goal of qualitative descriptive studies is to present a comprehensive summary of events in the everyday terms of those events and it entails a kind of interpretation that is low‐inference and does not require researchers to move as far from or into their data (Sandelowski, 2000).

### 3.3 Data collection

The study was conducted in two NHS hospitals in the South East of England. The EOMII scale asks participants to respond to each of 58 items using a four‐point rating scale, but limits their ability to express in detail their views regarding their work environment. To address this major weakness of survey questionnaires (Bowling, 2005), a large space was provided by the researchers at the end of the EOMII scale asking respondents to: “Finally, please add any comments you may have about your ward/work environment.”

This question could be described as unstructured (Bowling, 2005) because it was intended to give participants the opportunity to offer their perceptions of their work environments in their own words. O'Cathian and Thomas (2004) described the habitual “any other comments” at the end of structured questionnaires as a general open question which has the potential to elaborate responses to closed questions, allowing respondents to identify new issues not captured in the closed questions. Ignoring this data is potentially unethical and it has been recommended (O'Cathian and Thomas, 2004) that researchers should not ask open questions unless they are prepared to analyse the responses.

### 3.4 Strategy used in increasing the response rate

To increase the response rate, nurses on the wards were made aware of the study at ward meetings and an A3‐sized research poster was displayed on the notice board in each ward. The aims of the study were explained in the covering letter. One of the researchers visited each ward twice a week, over the course of the study, to check for responses, answer any questions and provide extra copies of the EOMII scale to the Registered Nurses who had lost or misplaced theirs. Reminder letters were sent 6 and 14 weeks after copies of the EOMII scale were first distributed.

Seventy‐five out of 247 participants (30%) who completed the EOMII scale wrote comments about their work environment. It is worth considering the potential effect of non‐response bias. It was possible that nurses who made comments about their work environment were more likely to be unhappy about the condition of their work environment and those least likely to comment were happy or indifferent about their work conditions. Although the response rate was low, it was the strength of feeling expressed by a small number of respondents around issues in their work environment that prompted the formal analysis of these data.

### 3.5 Sample

There was an even split of respondents across the two hospitals in the study; 37 from Hospital A and 38 from Hospital B. Most respondents were females (*N* = 70), with ages ranging from 20–≥60 years. Fifty‐five were Staff Nurses, 19 were Sisters and one was a Charge Nurse. Table 1 presents the role description of participants. Twenty‐seven were educated to degree level, while the remaining had a Diploma level qualification; years of nursing experience ranged from 1 month–40 years. Analysis of data revealed that there is no difference between the nurses with more years of working experience and the nurses with less working experience in terms of viewing their working environment.

**Table 1:** Role description of participants

Role	Description
Staff nurse	The basic grade of qualified nursing staff, who are involved in direct patient care.
Ward sister	A female nurse who has moved on to a higher rank/grade from a staff nurse, and has lesser responsibility to the ward manager. She has specific responsibilities for the running of the ward, in charge of nurses and involved in direct patient care.
Charge nurse	A male equivalent role of a ward sister.
Care Support Worker (CSW)	Care Support Worker, also called Health Care Assistants in some hospitals in the UK, are unlicensed/unregistered health personnel who work alongside nurses, midwives, doctors, and allied health professionals in looking after the general well‐being of patients.

### 3.6 Ethical considerations

The study received ethics approval from London‐Surrey Borders NHS Research Ethics Committee, study reference number 11/LO/1329. Participants’ anonymity and confidentiality were protected (see our earlier study).

### 3.7 Data analysis

It was presumed that the 75 participants who wrote comments about their work environment would have responded to the stimulus provided by the items of the eight attributes measured by the EOMII scale. Examination of the comments revealed that data were not organized in a pre‐defined manner. Their comments went beyond the scope of the EOMII scale, addressing issues such as staffing numbers, increasing workload, high stress levels and work engagement.

Inductive content analysis was used to analyse the data and it is a highly flexible method, which is applicable to a wide variety of different kinds of unstructured textual information (Bryman, 2016). The manifest content of the data, which are those elements that are physically present, countable and describe the content (Berg & Lune, 2012), was analysed. The key contents of the data were categorized, without the use of any specialist software, following the process of open coding, creating categories and abstraction, as described by Elo and Kyngas (2008). The data were actively read and initial ideas were noted and the data were actively searched for meanings and patterns and were given codes to describe all aspects of the content (Burnard, 1991,1996; Elo & Kyngas, 2008), some of which are shown in Table 2.

**Table 2:** Open coding (an illustration)

Participant codes	Comments	Open codes
P1012(OT/SR:40y)	Good environment. Good staff who all practice to their best. An excellent team and ward manager Xxx very supportive	Good work environment, good staff, practice to their best, excellent team, ward manager supportive
P1019(CD/CN:18y)	The ward team works well together, but this is not always acknowledged by senior mgt. A thank you from management can go a long way.	Good teamwork, lack of acknowledgement
P1026(EM/SN:25y6m)	This ward is an elderly acute medical ward, with patients who have dementia/confused. The staffing levels could be better, as you don't feel like a nurse.	Need for better staffing levels, low staff morale
P1036(GS/SN:6y)	We work very hard and have to deal with a high work load and we are short staff—the work is hard and we try to help our patients to the best of our ability.	Working hard, high workload, short staffed, hard work, pressure to help patients, helping the patients.
P1101(GS/SN:31y)	Our ward is extremely busy and high dependency, we are under constant pressure, day and night, to create empty beds, by transferring patients to other unsuitable wards, when not suitable for patients. Our staff has very high standards, but are pushed for time to care for patients by added paper work, check charts & cleaning tasks, admission documentation involves over 23 pages. The trust wastes so much paper with various checking charts that must be completed—we feel “do we look after patients or do cleaning & paper work!”	Extremely busy ward, constantly under pressure to create beds, patient safety compromised, not enough time to care, added paperwork, wasting papers, chart checking, cleaning tasks, unsure of nurses’ tasks.
P2077(ST/SN:10y)	Recent financial constraints are causing problems and bed pressure is reducing patient care.	Financial constraints causing problems, bed pressure reducing patient care
P2108(GS/SR:19y)	Working as a nurse in charge performing to coordinate the whole ward and at the same time looking after 6–10 in‐patients enabling delay and hinders provision of quality patient care.	Nurse in charge looking after 6–10 patients, delay, inhibits quality patient care
P2111(GS/SR:11y11m)	Often short staffed still expected to deliver high standard of care. I recently updated my skills in stoma care completing a course that lasted 6 days in total. This had to be done in my *own *time. The manager never gives positive feedback or encouragement so left feeling unappreciated and devalued	Often short staffed, high standard of care expected, attended a course in own time, manager never gave encouragement or positive feedback, left the job
P1070(RP/SR:5y9m)	Has been difficult recently—4 Ward Managers within last 12 months	Difficult situation, high turnover
P1071(RP/SN:3y9m)	Poor skill mix with retention problems. Unsupported by other staff which increases work load and leads to unproductive ward.	Poor skill mix, retention problem, unsupported, high work load, unproductive ward
P2047(RP/SR:11y)	Trust seems to care about the day to day bed capacity on the wards and not how challenging it is to give good quality care along with other pressures that ward staff encounter.	Bed management taking priority over patient care, quality care under pressure.
P1045(RP/SR:4y3m)	A busy ward where it can sometimes be stressful to work. Well supporting ward manager. Very experienced RNs. Good team to work in.	Busy ward, stressful to work, supportive ward manager, very experienced RNs, good team.

**Figure 2:**
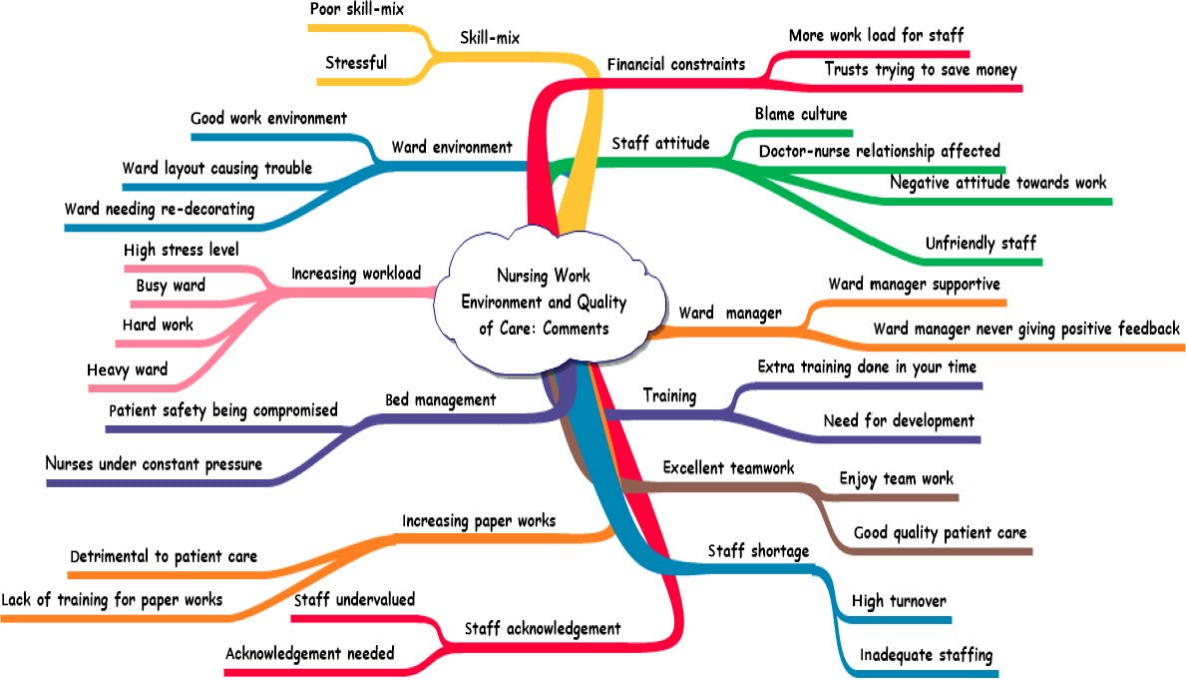
Creating categories phase

Following open coding, words and phrases were grouped together and “reduced” through a process of crossing out repetitions and similar words and phrases to produce a list of headings that accounted for all of the data in the transcript (Burnard, 1996). Some initial codes developed into the dominant themes, while others formed subthemes; for example, *ward manager* formed a dominant theme, with *ward manager supportive* and *ward manager never giving positive feedback* forming subthemes (see Figure 2).

**Figure 3:**
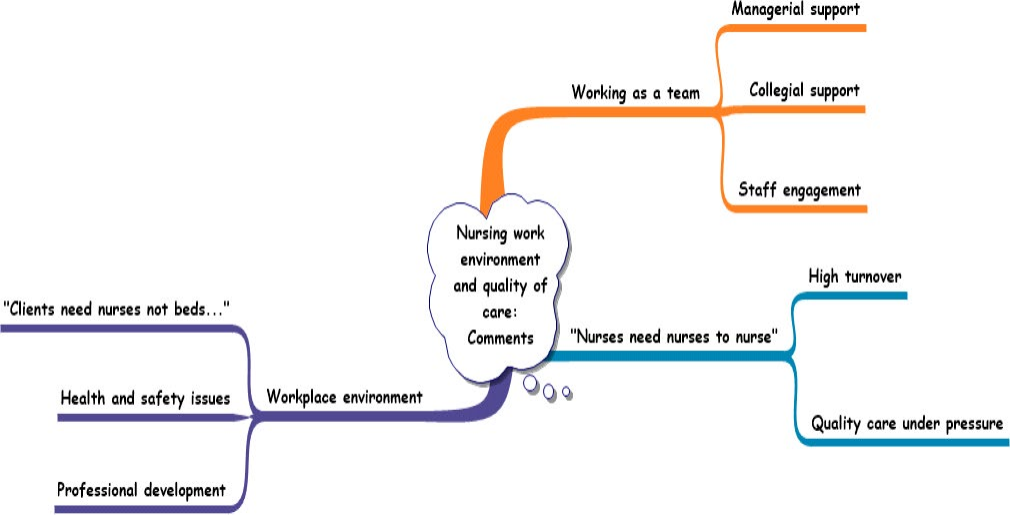
Abstraction phase/overview of themes and subthemes

During the abstraction phase, based on semantic and conceptual similarity, themes and subthemes were further condensed and given names that described their contents (Elo & Kyngas, 2008). For example, the themes *staff acknowledgement*, *staff attitude*, *excellent teamwork* and *ward manager* (Figure 2) were further condensed into a generic theme *Working as a team* (Figure 3); with *managerial support*, *collegial support* and *staff engagement* forming associated subthemes. Likewise, *staff shortage*, *increasing paperwork*, *increasing workload* and *skill mix* were condensed to form the generic theme “Nurses need nurses to work”, with *High turnover* and *Quality work under pressure*, as subthemes (Figure 3). Following this final refinement, three themes and eight subthemes were identified.

The final themes were allocated different colours and marked with corresponding fluorescent marking pens. The transcripts were then marked with different colours that corresponded to the themes and subthemes to which they belonged. Various coloured sections were then cut and pasted in their categories onto pages of A4 papers, giving a complete set of pages containing all of the analysed transcript, as recommended by Burnard (1996).

### 3.8 Rigour

Three authors independently went through the transcripts and identified similar themes, giving internal validity, reliability and credibility to the findings (Burnard, 1991). Content analysis is easily replicable and is often referred to as an objective method of analysis due to this transparent nature (Bryman, 2016).

## 4 FINDINGS

Three key themes and eight subthemes were identified: “nurses need nurses to nurse,” working as a team and workplace environment, as illustrated in Figure 3. Codes were used after each participant's quotes to represent information relating to their specialities, designations and years of nursing experience. Examples are presented in Table 3.

**Table 3:** Participants’ codes

Code	Interpretation
P1003(GM/SN:8y8m)	Participant 1,003, General Medical Ward, Staff Nurse, 8 years and 8 months of nursing experience
P1015(OT/SN:14y)	Participant 1,015, Orthopaedic Ward, Staff Nurse, 14 years of nursing experience
P1019(CD/CN:18y)	Participant 1,019, Cardiology Ward, Charge Nurse, 18 years of nursing experience
P1026(EM/SN:25y6m)	Participant 1,026, Elderly Medical Ward, Staff Nurse, 25 years and 6 months of nursing experience
P1064(GY/SN:4y3m)	Participant 1,064, Gynaecology Ward, Staff Nurse, 4 years and 3 months of nursing experience
P1101(GS/SN:31y)	Participant 1,102, General Surgical Ward, Staff Nurse, 31 years of nursing experience
P2042(HM/SN:1y9m)	Participant 2,042, Haematological Ward, Staff Nurse, 1 year and 9 months of nursing experience
P2047(RP/SR:11y)	Participant 2,047, Respiratory Ward, Ward Sister, 11 years of nursing experience
P2077(ST/SN:10y)	Participant 2,077, Stroke Ward, Staff Nurse, 10 years of nursing experience

### 4.1 “Nurses need nurses to nurse”

“Nurses need nurses to nurse” is a direct participant comment and captures participants’ understanding that staffing issues resulted in an inability to provide high quality care to patients. A vicious cycle existed where high turnover rates of staff on their wards resulted in inadequate staffing. Shortage of staff resulted in high patient to nurse ratios which negatively affected quality of care. Participants also associated these staffing issues to increased workload. They described the negative effects that this had on their physical and psychological well‐being, leading to staff having high stress levels. Participants expressed frustration and annoyance at having to compromise quality of care because they did not have sufficient staff on the wards. These issues are elaborated under two subthemes.

#### 4.1.1 High turnover

Participants revealed their struggles in trying to improve care delivery on their wards in the face of high turnover in the hospitals: “…We as a ward have a high turnover and are constantly trying to improve care delivery” [P1021(CD/SN:3y11m)]. One participant was specific about the rate of staff turnover stating: “…Has been difficult recently – 4 Ward Managers within the last 12 months” [P1070(RP/SR:5y9m)]. Another participant attributed poor skill mix to retention problems: “…Poor skill mix with retention problems…” [P1071(RP/SN:3y9m)].

Participants also stated that inadequate staffing led to each nurse being assigned a large number of patients which had negative impact on the quality of care that staff, patients and relatives experienced: “…14 for one nurse is too much. And if you are the nurse in charge you have to look after everyone i.e. patients, staff and even relatives” [P2072(EM/SR:18y)]. According to participants, staff shortage placed increased pressure on nurses, leading to low staff morale: “…when we are short staffed – this puts pressure on everyone and reduces staff morale” [P2112(GS/SN:5y)]. In addition to staffing issues, participants further highlighted that their well‐being and ability to function effectively were being compromised, as described below.

#### 4.1.2 Quality care under pressure

Participants highlighted their experiences of increased workloads, which in turn led to being stretched to their limits: “…staff stretched to limits, work load very high and staff here work to the limits…” [P2048(RP/SN:5y)]. As a result of high workload, which is a direct consequence of staff shortage, participants found it difficult to provide good quality care to their patients: “We…have to deal with a high work load and we are short staff – the work is hard and we try to help our patients to the best of our ability” [P1036(GS/SN:6y)]; and “…Due to the nature of the ward often I feel there is too much going on to give really good care to patients” [P2100(GM/SN:5m)]. Participants’ inability to provide high quality care to their patients was seen as more of a challenge when nurses were tasked with the dual duty of coordinating the whole ward and simultaneously looking after patients: “Working as a nurse in charge performing to coordinate the whole ward and at the same time looking after 6–10 in‐patients enabling delay and hinders provision of quality patient care” [P2108(GS/SR:19y)].

Apart from the busy nature of the nursing work, extensive attention was drawn to issues around increasing work demands attributed to age‐related comorbidity, rising complexity and acuity of the patients. Specifically, participants stressed the complexity of care needs related to those patients who were generally older, with mental health issues. According to the participants, the care of this category of patients was being compromised due to inadequate staffing and increased workload: “This ward is an elderly acute medical ward, with patients who have dementia/confused. The staffing levels could be better, as you don't feel like a nurse” [P1026(EM/SN:25y6m)]. In arguing for the increased work demands, especially regarding older patients and those with acute mental/health issues, one participant expressed belief that the Trust increased workload to save money: “Staff are given too many jobs to save money” [P1100(GS/SR:9y).

Concerns were also raised by participants over the increasing amount of burdensome paperwork, such as chart checking and admission documentation, which they felt had taken priority over patient care. One participant was not sure if nurses were needed to look after patients or do cleaning and paperwork: “Our ward is extremely busy…Our staff have very high standards, but are pushed for time to care for patients by added paper work, check charts & cleaning tasks…we feel “do we look after patients or do cleaning & paper work!”…” [P1101(GS/SN:31y)]. The increasing demand for documentation which takes participants away from bedside care prevents them from delivering high quality of care to their patients: “Very busy ward with not enough staff to meet the needs of our patients …an ever increasing amount of paperwork. Registered Nurses need to be “at the bedside”…” [P1110(GS/SN:37y)].

Participants voiced concerns over their physical and psychological well‐being. Nurses perceived their work environment as being stressful and were worried about their inability to give quality care to patient as a result of staff shortage, increasing workload and pressure, leading to exhaustion, burnout and stress: “Due to staffing levels and pressure I feel we are not able to give the quality of care we would all want to give. Staff feel burn out and stressed” [P2049(RP/SN:4y10m)].

In addition to individual staffing issues highlighted above, participants also indicated that teamwork significantly influenced the quality of their work environment.

### 4.2 Working as a team

Participants identified teamwork as a source of support in their work environment. Many participants placed emphasis on teamwork being demonstrated in staff members’ professional relationship with one another. They described teamwork as one of the facilitating aspects of their work environment that they considered essential to improving their work experiences, as well as supporting them in providing quality patient care. Professional relationships linked to teamwork included ward manager support and support from other members of staff. They also highlighted specific inhibiting factors in teamwork, such as the absence of collaborative doctor–nurse relationships and negative attitudes of some nursing staff. These factors are elaborated under three subthemes.

#### 4.2.1 Managerial support

Many of the participants made positive comments about their ward managers. They described the supportive role of their ward managers as facilitating nursing practice. Supportive behaviours of the ward manager were identified as “understanding,” “helping to improve confidence,” “approachable,” “accessible,” “pleasant,” “good” and “very good.” Typical responses were as follows: “…Ward manager is very good and approachable…” [P2015(OT/SR:18y6m)], “…an experienced ward manager. She is very accessible and pleasant” [P1074(RP/SN:27yr)] and “…So far it's getting better here due to good management of our new manager” [P1015(OT/SN:14y)]. Participants’ descriptions of their excellent or committed team, as one which is enhanced by their ward managers: “…Good staff who all practice to their best. An excellent team and ward manager Xxx very supportive” [P1012(OT/SR:40y)], “… We have a committed team run by 2 supportive managers…” [P1104(OT/SN:22y4m)]; and “N Ward is a fantastic place to work due to management of the ward…” [P2067(CD/SN:11m)].

Participants stated that due to the supportive role of their ward managers, their confidence improved. For example, one participant emphasized the understanding nature of the ward manager: “I have got a wonderful ward manager who has helped me to improve in myself and my confidence because of her understanding nature” [P2057(GM/SN:3m)]. Participants described the supportive role of the ward manager as contributing to enhanced teamwork and better work environment. They indicated that at times when there was tension between staff and the ward got busy and stressful, the supportive role of the ward manager helped to alleviate the unfavourable work situation, thereby enhancing patient care: “…busy ward where it can sometimes be stressful to work. Well supporting ward manager…” [P1045(RP/SR:4y3m)] and “Although at times, there is tension between staff. We work well as a team and provide excellent care to patients. Ward manager supports the ward” [P2042(HM/SN:1y9m)].

Out of the 14 participants that commented about their ward managers, most (*N* = 12) were complimentary; however, two participants emphasized the lack of interpersonal relationship and the managers’ inability to give encouragement or constructive feedback, leading to staff members feeling unappreciated and undervalued. The participants also revealed that those ward managers were instrumental to their resignation from the ward: “…The manager never gives positive feedback or encouragement so left feeling unappreciated & devalued” [P2111(GS/SR:11y11m)]. The above comment was echoed by another participant who revealed that the attitude of the ward manager led to his resignation. As the participant stated, the ward manager's attitude destroyed the potential for the ward to shine. However, this participant stated that staff nurses were supportive towards each other: “Staff nurses support each other very well but the ward manage…her attitude towards staff is combative and usually aggressive. This has destroyed any potential for the ward to shine. Hence I have recently left…” [P2135(MW/SN:1y)].

Participants moved from talking about the ward‐level managers (i.e., the ward managers) to the managers at board of hospital director level who are outside the wards (known in the UK as Trust‐level managers, or management, or senior management), as they have different perceptions about the two levels of management. One participant identified the lack of support as stemming from the Trust rather than the actions of the ward manager and colleagues: “I enjoy working with my colleagues and our Ward manager as we work as a team. However, I feel we do not get the right support and back up from the Trust” [P1091(RP/SN:3y10m)]. The lack of appreciation or acknowledgement for their team efforts from management made them feel undervalued: “The ward team works well together, but this is not always acknowledged by senior mgt. A thank you from management can go a long way” [P1019(CD/CN:18y)]; and “Our involvement as nurse is not appreciate enough…” [P1064(GY/SN:4y3m)].

The presence of blame culture in the hospital was revealed as a factor that could inhibit participants from putting their best performance to team efforts: “…but feel there is a culture of blame…” [P2125(GM/SN:6y)] and “A hospital with blame culture, not good…” [P2072(EM/SR:18y)]. In addition to management, the success of working as a team rests in the degree of support received from the nurses’ immediate colleagues.

#### 4.2.2 Collegial support

Many of the participants indicated that the level of support received from other members of staff through teamwork increased their sense of belonging and strengthened their relationship with colleagues: “…staffs are competent, friendly, enthusiastic with a positive attitude towards patients care and with colleagues. Team work is always present among colleagues” [P2094(GS/SN:17y1m)]. The ethos of collegial support was echoed by other participants from their comments such as: “…my colleagues are really friendly, supportive…” [P2103(ST/SN:7y6m)], “… fellow colleagues working so well as a team and their support” [P2067(CD/SN:11m)], “Staff nurses support each other very well…” [P2135(MW/SN:1y)]; and “Very supportive and staff work well together in a team” [P2123(GM/SN:1y)].

However, one participant provided a contrasting view from those who were in praise of collegial support. This participant indicated that retention problems and increased workload, which led to an unproductive ward, were direct consequences of being “…Unsupported by other staff which increases work load and leads to unproductive ward…” [P1071(RP/SN:3y9m)]. In the same vein, some participants applauded the supportive role of their colleagues and ward managers, but nevertheless highlighted the rude behaviour of some of the staff as an inhibiting factor to effective patient care: “… The support I have had as a newly qualified nurse has been more than I could have anticipated, received from staff nurses, clinical sisters and the ward manager alike…the clinical support workers are amazing…The only negative comment I have to make is that I find some specialist nurses who work at times on the ward can be rude and treat nurses on the ward as if they have less value in contributing to that particular area of care” [P2107(GS/SN:4m)].

The importance of inter‐professional relationships in the provision of high quality care was also emphasized by the participants. They indicated that although they had excellent teamwork and good collegial relationships that were enhanced by managerial support, a collaborative nurse–doctor relationship was missing. For example, a participant linked inadequate staffing to the breakdown of nurse–doctor relationships, leading to loss of important information, affecting patient care: “…Due to less nurses on the ward, the relationships between Drs & SN can be affected and important information can often be omitted” [P2113(GS/SR:7y11m)]. Another participant also supported the view that good inter‐professional relationships were important in facilitating high quality care: “The CSWs [Care Support Workers; also called Health Care Assistants, are unlicensed/unregistered health personnel who work alongside nurses, midwives, doctors and allied health professionals in looking after the general well‐being of patients] working particularly hard on this ward they are an asset…Doctors‐nurse relationship very poor but other MDT [Multidisciplinary Team] members work really well together as a team and have a shared approach to patient care…” [P2015(OT/SR:18y6m)]. Although participants highlighted the presence of cohesiveness among the nurses, demonstrated through hard work as a team, they also drew attention to how frustrating it was to work alongside colleagues who were evidently not interested in their jobs, or committed to the team.

#### 4.2.3 Staff engagement

Participants expressed frustration over some nurses’ poor motivation and lack of interest on the job. According to the participants, being absorbed in and being enthusiastic about their job would contribute to patient care, team cohesion, as well as improving their relationship with other members of staff. Some participants expressed disillusionment when they observed others displaying lack of interest in the job, often at the expense of good quality care to patients: “…Some staff are just frustrating to work with. Other staff feels/thinks they know everything and does not want to be told what to do, but they don't do things the way it should be done. Other staff goes to work because they have to, no interest or enthusiasm…” [P2072(EM/SR:18y)].

In contrast to the above comments, many of the participants acknowledged their wards as good working environments where they were either happy or enjoyed working: “I am happy to work here as a nurse …” [P2103(ST/SN:7y6m)]; and “I enjoyed working in my workplace since I started here” [P1102(EM/SN:11yr1m)]. In addition, some participants enjoyed their jobs and would be happy to have their family members admitted on their wards: “As a nurse, I enjoy and am proud of the level & quality of care my ward gives, that I would be happy for any family member to admitted to *my* ward.…” [P2125(GM/SN:6y)].

Some participants also commented that even though working on their wards could sometimes be stressful, they still enjoyed coming to work due to the workplace culture: “…I enjoy coming to work sometimes it is a bit stressful but we also have our good days” [P2119(GS/SN:2y)]; and “Stressful but fun and engaging + challenging…” [P1035(GS/SR:14y)]. These comments indicated that the participants were engaged with their jobs. In addition to inadequate staffing and teamwork, the workplace environment itself, in the form of pressure to create beds, health and safety issues and professional development, was identified as having impact on their work and quality of care given to patients.

### 4.3 Workplace environment

Participants expressed worries over their workplace environment in terms of the physical work space and the need for further training and development. They were concerned that the absence of specific features such as adequate ventilation in their work environment could have an impact on health and safety of both the staff and the patients. They were also concerned over the priority the management placed over bed management.

#### 4.3.1 “Clients need nurses not beds…”

“Clients need nurses not beds…” is a direct participant comment and captures participants’ understanding that the quality of care given by nurses is not about the physical resources. Rather, it involves how well trained or skilled the nurse is and the availability of the nurse. Participants believed that the focus of the Trust was on bed management, not on ensuring that patients were given the best care. According to the participants, bed managers continued to mount pressure on the nurses to create empty beds when it was inappropriate to discharge current patients, a situation the participants believed was detrimental to patient care. Nurses reported that they do not feel “listened to” by bed managers: “…feel we are not listened to by bed management who “bed” our query homes, even though they may not go home. When we have no beds we do actually mean we have no beds!” [P2025(GY/SN:6m)]. Other participants elaborated on how bed management and bed pressure were taking priority over patient care: “Trust seems to care about the day to day bed capacity on the wards and not how challenging it is to give good quality care along with other pressures that ward staff encounter” [P2047(RP/SR:11y)]; and “Recent financial constraints are causing problems and bed pressure are reducing patient care” [P2077(ST/SN:10y)]. This issue of improper bed management resulted in compromised patient care when, consequently, patients were admitted into inappropriate wards: “Our ward is extremely busy and high dependency, we are under constant pressure, day and night, to create empty beds, by transferring patients to other unsuitable wards, when not suitable for patients…” [P1101(GS/SN:31y)]. Furthermore, participants identified that apart from the pressure they were facing to create beds, the physical environment of their wards was creating health hazards and potentially compromising the quality of care nurses could provide to patients.

#### 4.3.2 Health and safety issues

Participants highlighted that the physical condition and appearance of their wards could constitute health and safety issues. For instance, lack of ventilation in the ward was an issue: “Too hot – airless – troubled by outside noise – car/ambulance fumes…“ [P2026(GY/SN:37y10m)]; and “Hot – cramped – airless – windowless…” [P2133(GY/SN:38y2m)]. Participants also expressed their dissatisfaction with the lack of repair and maintenance of hospital equipment and furniture: “…Repairing of bed side lights are almost impossible” [P1059(GY/SN:8y6m)]; and: “…The ward itself is old and needs decorating…” [P2015(OT/SR:18y6m)]. Nevertheless, despite the stated shortcomings of the ward environment, one participant was of the view that she worked in a “Good ward and environment, also well organised” [P2003(EM/SR:20y)]. Finally, participants identified that apart from the physical environment, their work was influenced by workplace practices consisting of policies, which guide the provision of continuous professional development courses for nurses.

#### 4.3.3 Professional development

Participants emphasized the importance of workplace practices such as professional development for the performance of particular tasks, which have an impact on patient care. They registered their dissatisfaction over the Trust's inability to meet their educational and training needs. Accessibility of these courses would have enhanced the fulfilment and performance of their roles. For example, a participant expressed the need for further training: “…The staff nurses need a lot of development…” [P2015(OT/SR:18y6m)]. Whilst one participant expressed dissatisfaction over the lack of encouragement and time for the completion of a six‐day course: “…I recently updated my skills in stoma care completing a course that lasted 6 days in total. This had to be done in my *own *time…” [P2111(GS/SR:11y11m)]; in contrast, a participant who was newly employed in the hospital nevertheless expressed satisfaction over the planned development and study days: “I am new at this trust. I am very happy about the development I am getting and all the study days planned for me” [P1003(GM/SN:8y8m)].

Participants in this study provided an insight into factors which affected the quality of their work environment and how these factors influenced their ability to provide quality care.

## 5 DISCUSSION

In response to the invitation to make comments about their work experiences, many nurses offered additional insight into aspects of their work environment which gave them concerns. This paper is the first to identify that despite the staffing problems that nurses faced and the resultant high workload and stress they were experiencing, nearly all the participants who commented about their ward managers, made positive comments. In describing their ward managers, most participants used positive words such as “approachable,” “accessible” and “pleasant”. Staff nurses in an American study by Schmalenberg and Kramer (2009) identified similar behaviours of the nurse manager as essential for a healthy work environment. Some behaviours of nurse managers identified as most supportive were being accessible, approachable, promoting staff cohesiveness and providing both positive and negative feedback (Schmalenberg & Kramer, 2009).

A study of nurses in acute hospitals in London demonstrated the importance of the ward manager in the effective functioning of the ward and a key determinant in nurses’ decisions to leave or to remain in the job (Barron, West, & Reeves, 2007). To facilitate this role, the ward manager needs to be seen as supportive, approachable and accessible, to improve teamwork, strengthen staff engagement and enable staff to practice effectively. The ward manager occupies a pivotal role in the effective operation of the ward because of his/her good knowledge of the ward. Ward managers are also placed in a position of advantage to enhance nurses’ performance because they have direct contact with the nursing staff. Numerous large studies (e.g., Kramer et al., 2007; Squires, Tourangeau, Laschinger, & Doran, 2010; Cummings et al., 2010; Ritter, 2011) have demonstrated a robust link between leadership behaviour in the work environment and the nursing workforce.

Only one participant (with four months working experience) commented negatively on the behaviour of specialist nurses, noting their behaviour as rude and condescending. It could be argued that the staff nurse could be experiencing what Kramer, Brewer, and Maguire (2013: 348) described as “reality shock” a concept used to describe the reactions of newly qualified staff nurses when they found out that the university and hospital cultures are vastly different. This occurs when they find themselves in a work situation for which they have spent several years preparing and for which they thought they were going to be prepared and then suddenly find out they are not. This situation can be disorientating leading to low job satisfaction or attrition.

Findings from this study indicate that many nurses working in the two hospitals faced huge challenges in their work environment with extensive emphasis placed on staffing shortage, having huge impact on their ability to provide high quality care. They also identified that financial constraints in the organization made alleviation of staff shortages very difficult. Participants in Kieft, Brouwer, Francke, and Delnoij (2014) also indicated that management was tied to a system that was dominated by controlling costs. Inadequate staffing in the current study resulted in high stress levels and increasing workload. Choi, Pang, Cheung, and Wong (2011) also described inadequate staffing and absenteeism as having formed a vicious cycle, that is, nurses unable to cope with work pressure are absent from work, which in turn increases the workload of the remaining staff, who gradually develop an inclination to leave their current posts, leading to increasing voluntary turnover.

This research provided the opportunity for nurses to express their feelings about factors inhibiting high quality care and teamwork, such as the presence of blame culture, lack of staff appreciation/acknowledgement, bed management and inadequate staff development. Comments made by the participants may imply that no one was listening to the nurses and that they have very little executive power to influence or change situations in the hospitals. A report by the King's Fund (Ham, 2014) has described the NHS as a service characterized by emphasis on reforms, driven from the top down by politicians and regulators, as it is centrally controlled and funded through general taxation (Buchan, 1994; Klein, 2012). It has also been highlighted (Francis, 2013; The King’s Fund, 2012) that NHS leaders focused more on the delivery of targets than engaging patients and staff. The NHS Improvement (2016) has developed a framework that supports a more participative decision‐making style in the NHS, similar to the Magnet model (ANCC, 2017) in the United States. This participative decision‐making style is now becoming more evident in the NHS. For example, a report by Stephenson (2017) highlighted that the Barts Health NHS Trust in England is planning to set up a new group or clinical senate to include nurses at all levels for the purpose of strengthening the voice of frontline nursing staff by collecting their views. This clinical senate will identify factors that are hindering good care or changes they think are needed and identify potential solutions. It could be argued that the formation of this clinical senate is a move towards a more parallel channel of communication to facilitate interactions between ward managers, policy makers and the frontline nurses in the NHS.

### 5.1 Limitations

It is acknowledged that the data gathered were short comments from the participants and this method of data collection did not present the researchers any opportunity to probe the participants or to clarify information given. In addition, this study was conducted in two NHS hospitals in the South East of England; findings may not be typical of all acute trusts in England, it may therefore limit generalization of the study.

## 6 CONCLUSION

Findings from this study have provided better understanding of the challenges experienced by nurses in their work environment, particularly in terms of constraints on their ability to provide high quality patient care. These constraints are currently largely beyond the resources of nurses themselves to address. The practice and policy implications of this research are for nurse leaders and policy makers to involve ward nurses in decision and policies for practice, to ensure effective delivery of nursing care. Further work on adapting the EOMII for use in England might focus on incorporating questions about structural elements of NWE. At the moment, the EOMII focuses exclusively on the “process” element of Donabedian's Structure–Process–Outcome model of the determinants of quality and it may have been the absence of attention to structural aspects that prompted so many nurses to give their comments.

## CONFLICT OF INTEREST

No conflict of interest has been declared by the authors.

## AUTHOR CONTRIBUTIONS

All authors have agreed on the final versions and meet at least one of the following criteria (based on those recommended by the ICMJE*): (a) substantial contributions to conception and design of, or acquisition of data or analysis and interpretation of data; (b) drafting the article or revising it critically for important intellectual content. *http://www.icmje.org/recommendations/browse/roles-and-responsibilities/defining-the-role-of-authors-and-contributors.html).
